# Yoga as a therapeutic approach to mental health in university students: a randomized controlled trial

**DOI:** 10.3389/fpubh.2024.1406937

**Published:** 2024-06-05

**Authors:** Yolanda Castellote-Caballero, María del Carmen Carcelén-Fraile, Agustín Aibar-Almazán, Yulieth Rivas-Campo, Ana María González-Martín

**Affiliations:** ^1^Department of Health Sciences, Faculty of Health Sciences, University of Jaén, Jaén, Spain; ^2^Department of Health Sciences, Faculty of Health Sciences, University of Atlántico Medio, Las Palmas de Gran Canaria, Spain; ^3^Department of Education and Psychology, Faculty of Social Sciences, University of Atlántico Medio, Las Palmas de Gran Canaria, Spain; ^4^Faculty of Human and Social Sciences, University of San Buenaventura-Cali, Santiago de Cali, Colombia; ^5^Department of Psychology, Higher Education Center for Teaching and Educational Research, Madrid, Spain

**Keywords:** yoga, perceived stress, emotional wellbeing, anxiety, university students

## Abstract

**Objectives:**

The purpose of this study has been to analyze the efficacy of a yoga-based intervention on stress, emotional wellbeing, state anxiety and trait anxiety in university students.

**Methods:**

This study was a randomized controlled trial. The sample consisted of 129 university students, of which 65 underwent a yoga training for 12 weeks and a control group that did not carry out any type of intervention. The primary outcome was stress, which was collected through The Perceived Stress Scale (PSS). The secondary outcomes were emotional wellbeing which was measured through the Warwick-Edinburgh Mental Wellbeing Scale (WEMWBS), and anxiety through State–Trait Anxiety Inventory (STAI). All variables were measured before and after the intervention.

**Results:**

Participants in the experimental group showed statistically significant improvements in the primary outcome perceived stress (Cohen’s *d* = 0.44) and the secondary outcomes emotional wellbeing (Cohen’s *d* = 0.47), and both state anxiety (Cohen’s *d* = 0.38) and trait anxiety (Cohen’s *d* = 0.80) compared to the control group that did not carry out any type of physical training.

**Conclusion:**

This study demonstrated that a 12-week yoga intervention can significantly reduce perceived stress and anxiety, and improve emotional wellbeing in university students. Initially, the experimental group (EG) reported higher levels of stress and anxiety than the control group (CG), but after the intervention, the EG experienced significant improvements compared to the CG.

## 1 Introduction

In recent years, there has been a notable increase in interest toward the mental health of university students (1, 2), in response to the observed increase in the prevalence of psychological disorders in this population (3). Prior studies (4–6) have shown that many students encounter elevated levels of stress and anxiety throughout their university experience. While universities provide avenues for personal and academic development, they also present challenges that can impact mental wellbeing negatively (7). Stress, anxiety, and shifts in emotional health are increasingly recognized issues in this setting, affecting not only academic achievements but also the overall quality of life among young university students.

Perceived stress among university students can originate from various sources, such as academic workload, social expectations, financial pressures, and the transition to independent living at university (8). This constant tension can manifest in physical, emotional, and cognitive symptoms, affecting concentration, decision-making, and interpersonal relationships (9). Anxiety is also a common condition among university students (10) and manifests in two main forms: as a temporary state and as a persistent trait. The temporary state of anxiety is commonly experienced in moments of academic stress, such as during exams or presentations. However, these episodes are transient and tend to disappear once the stressful situation has passed. On the other hand, anxiety as a trait involves a predisposition to experience anxiety in various situations, without depending on a specific stressful stimulus (11). Students with this characteristic may show chronic worry, persistent muscle tension, excessive self-demand, and anticipatory fear about their academic and professional future (12), which can have a significant impact on their academic performance and on their quality of life both physically and mentally (13, 14).

Given this context, it is crucial to investigate effective interventions that can mitigate stress, reduce anxiety, and promote emotional wellbeing among the student population. Yoga, an ancient discipline that merges physical movements to strengthen and flexibilize the body, breathing exercises to control respiratory rhythm, deep relaxation methods to alleviate physical and mental tension, meditation to increase awareness of the body and mind, and mindfulness techniques to enhance emotional regulation through the practice of yama and niyama (15), has gained popularity as a potential strategy for improving mental health and overall wellbeing (16). From a scientific perspective, studies support the benefits of yoga in reducing mental pathologies (17–19). Yoga practices, which include mind–body interaction through physical exercise combined with breathing techniques and meditation, have shown an increase in noradrenaline levels and a reduction in plasma adrenaline levels (20), a decrease in cortisol levels and an improvement in the response of the autonomic nervous system (21), which translates into a reduction of perceived stress. Additionally, it has been observed that the practice of yoga increases the production of neurotransmitters like serotonin (22) and dopamine (23), associated with emotional wellbeing.

These findings suggest that yoga has proven effective in regulating various biological systems, including the autonomic nervous system, the hypothalamic–pituitary–adrenal (HPA) axis, and heart rate variability, which supports its beneficial effects on mental and emotional health (15). This makes it a valuable tool to incorporate into intervention programs aimed at helping university students manage stress, anxiety, and improve their quality of life. The present study seeks to analyze the effectiveness of a yoga-based intervention on stress, emotional wellbeing, state anxiety and trait anxiety in university students.

## 2 Materials and methods

### 2.1 Research design and participants

Randomized controlled trial aimed at analyzing the effectiveness of a 12-week yoga-based intervention on perceived stress, emotional wellbeing, state anxiety and trait anxiety in university students. This study was registered prior to its commencement (NCT06304662) and was approved by the Ethics Committee of the Mid-Atlantic University (CEI/05–006).

The recruitment of participants was carried out from November to February of 2024, taking advantage of placing advertisements at key points within the university campus, dissemination through social networks linked to the university, and direct contact with students via emails sent from the educational entity’s database. Those who showed interest in participating in the study completed an informed consent form in accordance with the Declaration of Helsinki, Good Clinical Practice, and all applicable laws and regulations, which clearly explained the study’s goals, the methods to be followed, potential risks and benefits, and the confidential handling of the obtained data. Included in the study were students who: (i) were university students who had participated in a yoga training program in the previous 12 months; (ii) have adequate physical capacity to carry out the activities demanded in the study; and (iii) that they have the ability to interpret the project instructions, programs and protocols. The exclusion criteria were: (i) that they did not meet the minimum number of sessions required to complete the study; and (ii) that they had extensive experience and experience in the practice of yoga.

### 2.2 Randomization

The distribution of participants was carried out using a sequence of random numbers generated by computer, placing individuals equally in an experimental group (EG) and a Control group (CG) following a 1:1 ratio. The group assignments were kept hidden from both the participants and the researchers, and the physiotherapist responsible for carrying out the intervention. This process was carried out using sealed, opaque envelopes that were sequentially numbered, which were kept locked and were only opened by a person not directly involved in the study. At the conclusion of this procedure, 65 individuals were assigned to the experimental group and another 65 to the Control group.

### 2.3 Intervention

Participants in the experimental group (EG) underwent a yoga program twice a week (Tuesdays and Thursdays) for 12 weeks in person, totaling 24 sessions with duration of 60 min each. Each of these sessions during this period consisted of a total of 4 distinct parts: (i) 5 min dedicated to breathing techniques through Pranayama postures; (ii) 10 min of warm-up based on joint movement exercises; (iii) 35 min allocated to the central part of the intervention where postures based on Hatha yoga were performed, one of the most traditional and widely practiced forms of yoga that focuses on physical postures (asanas), breathing techniques (pranayama) and meditation (dhyana); and (iv) 10 min for relaxation techniques based on flexibility and stretching exercises. In the core part of the intervention, different postures were carried out distributed according to position: (a) standing postures: Utkatasana (Chair Pose), Uttanasana (Standing Forward Bend), Vrksasana (Tree Pose), Garudasana (Eagle Pose), Naṭarajasana (Dancer Pose), Virabhadrasana I (Warrior I), Virabhadrasana II (Warrior II), Virabhadrasana III (Warrior III), Utthita Trikonasana (Triangle Pose), and Parivrtta Baddha Parsvakonasana (Revolved Side Angle Pose); (b) seated postures: Dasana (Staff Pose), Janu Sirsasana (Head-to-Knee Pose), Parivrtta Janu Sirsasana (Revolved Head-to-Knee Pose), Baddha Konasana (Bound Angle Pose), Gomukhasana (Cow Face Pose), Navasana (Boat Pose), and Ardha Matsyendrasana (Half Lord of the Fishes Pose); (c) knee postures: Balasana (Child’s Pose), Supta Virasana (Reclining Hero or Heroine Pose), Ustrasana (Camel Pose), Eka Pada Rajakapotasana (One-Legged King Pigeon Pose), Parighasana (Gate Pose), and Simha asana (Lion Pose); (d) prone postures: Savasana (Corpse Pose), Apanasana (Knees-to-Chest), Setu Bandha Sarvangasana (Bridge Pose), Sarvangasana (Shoulder Stand), Viparita Karani (Legs-Up-the-Wall Pose), Halasana (Plow Pose), and Jathara Parivrtti Asana (Belly Twist); and lastly, (e) supine postures: Bhujangasana (Cobra Pose), Dhanurasana (Bow Pose), and Salabhasana (Locust Pose).

All sessions were under the supervision of a professional trained in Physiotherapy with more than 5 years of experience in various styles of yoga. A criterion for analysis was set as participation in at least 80% of the scheduled sessions. Participants in the control group were recommended to continue with their daily lives without significant changes; especially they were instructed not to enroll in any training program or structured physical activity during the study period. The current levels of physical activity of the participants were controlled in both the intervention group and the control group through regular telephone calls. These calls were made periodically throughout the study period and served to collect detailed information about any physical activity in which the participants were engaged outside of the scheduled study sessions.

### 2.4 Outcomes

The general procedure for data collection included administration of questionnaires in a controlled environment, usually within the university campus, to ensure accuracy and consistency in responses. Participants completed these questionnaires under supervision to resolve any questions in real time and ensure complete understanding of each question. Regarding the schedule of evaluations, they were carried out the week before the start of the yoga intervention, thus establishing the baseline for the measurements. Subsequently, once the intervention was completed, the participants were evaluated again the following week. This scheduling of evaluations allowed immediate and effective monitoring of the effects of the intervention. Before assignment, descriptive characteristics such as age, sex, employment, physical activity, weight, height, and BMI were collected in the presence of experienced interviewers. The Body Mass Index (BMI) was calculated as weight (kg)/height (m^2^) (24).

Primary outcome:

Perceived stress was measured through The Perceived Stress Scale (PSS) (25) in its Spanish version (26), a 14-item questionnaire that assesses the level of stress perceived over the last month. It consists of 14 items with a response format on a five-point scale (0 = never, 1 = almost never, 2 = sometimes, 3 = often, 4 = very often). The total PSS score is obtained by reversing the scores of items 4, 5, 6, 7, 9, 10, and 13 (in the following way: 0 = 4, 1 = 3, 2 = 2, 3 = 1, and 4 = 0) and then adding up the 14 items. The direct score obtained ranges from 0 to 56 points and indicates that a higher score corresponds to a higher level of perceived stress.

Secondary outcomes:

Emotional wellbeing was measured through the Warwick-Edinburgh Mental Wellbeing Scale (WEMWBS) (27), a 14-item questionnaire on mental wellbeing that includes subjective wellbeing and psychological functioning. All items on a 5-point Likert scale are phrased positively and address aspects of positive mental health, measuring the frequency of the subject’s attitudes from “never” to “always.” Higher scores indicate better mental wellbeing. The Spanish version of WEMWBS showed good psychometric properties similar to the original scale from the United Kingdom (28).

The anxiety of the participants was measured by the State–Trait Anxiety Inventory (STAI) (29), a self-report composed of 40 items designed to assess two independent concepts of anxiety: state anxiety (a transient emotional condition) and trait anxiety (a relatively stable anxious propensity). The temporal frame of reference for state anxiety is “right now, at this moment” (20 items) and for trait anxiety is “in general, on most occasions” (20 items). Each subscale is made up of a total of 20 items in a 4-point Likert response system according to intensity (0 = almost never/none; 1 = somewhat/sometimes; 2 = moderately/often; 3 = very much/almost always). The total score on each of the subscales ranges from 0 to 60 points. Both the original questionnaire and its Spanish version have good psychometric properties, with levels of internal consistency ranging, for both the total score and each of the subscales, between 0.84 and 0.93 (30).

### 2.5 Sample size calculation

The sample size was determined using the freely available statistical software Epidat 4.2 (Xunta de Galicia. Consellería de Sanidade-Servizo Galego de Saúde) with the following parameters referring to the level of stress perceived as a primary outcome: it was proposed to evaluate an expected mean difference of 2.4 units between the two groups (31) or a reduction of 0.6 in each group (32). With a significance level of 5% (corresponding to a z-value of 1.96) and a power of 90% (corresponding to a z-value of 1.28). A loss percentage of 15% was also considered, which allowed adjusting the sample size to 122 participants to compensate for possible dropouts or missing data during the study.

### 2.6 Statistical analysis

For population characterization, categorical variables were presented in terms of frequency and percentage. An exploratory analysis was conducted to validate the normal distribution of quantitative variables, ensuring adherence to the requisite assumptions for each analysis (Kolmogorov–Smirnov >0.05). Subsequently, mean values and standard deviations were computed for each variable of interest. The Student’s *t*-test for independent samples was employed to assess inter-group differences.

Moreover, an effective treatment analysis was executed. This entailed the utilization of a mixed analysis of variance, with the intervention (YGE vs. CG) acting as the between-groups factor and the measurement time (pre-treatment versus post-treatment) serving as the within-subject variable. Dependent variables, such as perceived stress, emotional wellbeing, and anxiety, underwent separate analyses. An exploration of potential interactions between treatment group and measurement time was conducted.

Additionally, Cohen’s d was utilized to determine intergroup effect sizes, with values of ≤0.2, 0.5, and 0.8 indicating small, medium, and large effects, respectively. A significance level of *p* < 0.05 was established for all analyses. Bonferroni correction was used to adjust the *p*-values for independent samples t-tests and for multiple comparisons in the case of the mixed analysis of variance. Statistical computations were performed using SPSS statistical software, version 17.0 (SPSS, Inc., Chicago, IL, United States).

## 3 Results

**Figure 1:**
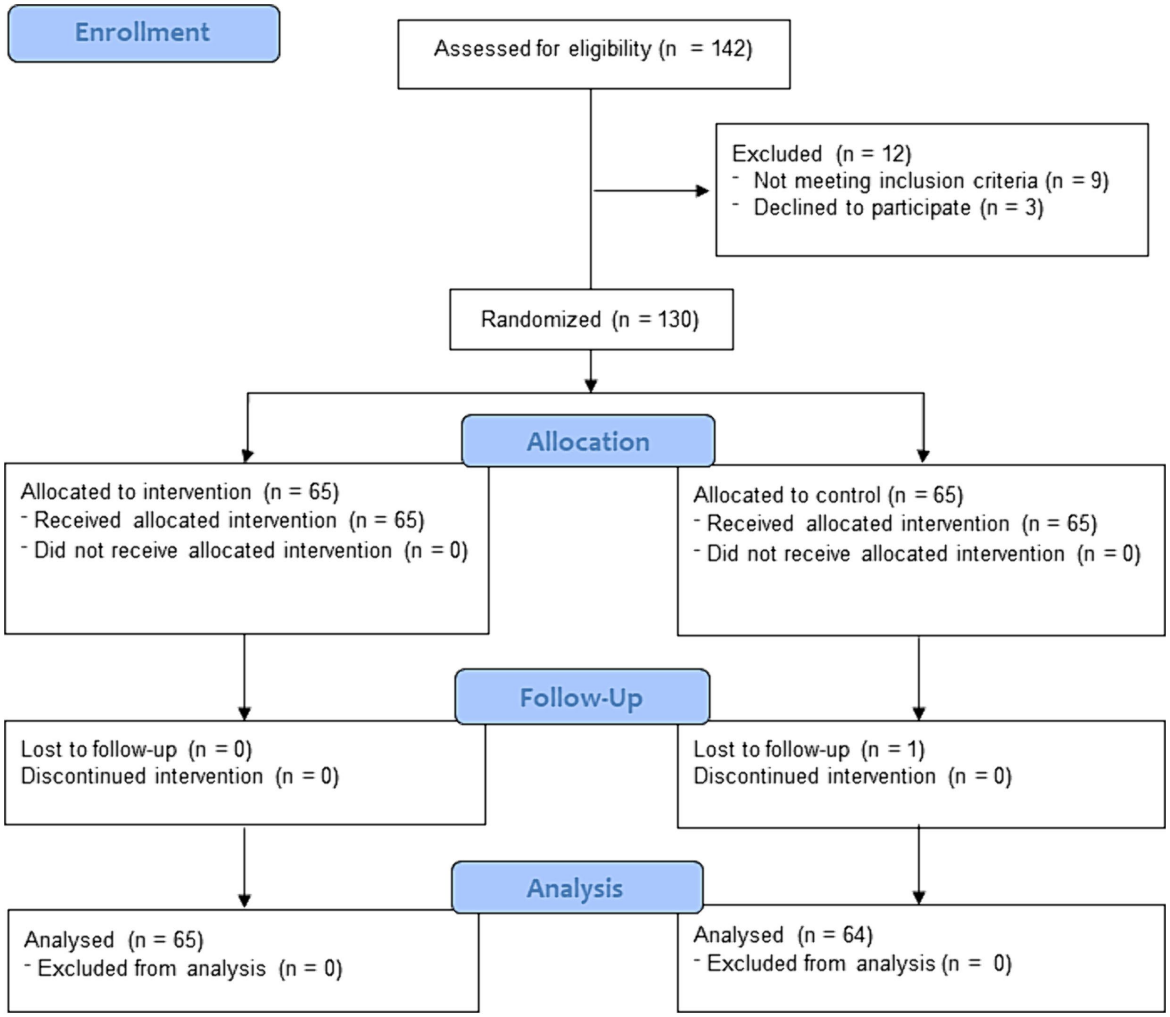
Flow diagram of the study.

At the start of the study, a total of 142 university students from various universities were contacted, of which 130 met the inclusion criteria and participated in the study (Figure 1). This study consists of 48.8% male participants and 51.2% female participants (Table 1).

**Table 1:** Initial characteristics of study participants.

		Total (*n* = 129)	Experimental group (*n* = 65)	Control group (*n* = 64)	*p-*value
Age		20.29 ± 1.77	20.29 ± 1.78	20.30 ± 1.76	0.487
Sex	Male	63 (48.8)	33 (52.4)	30 (47.6)	0.649
	Female	66 (51.2)	32 (48.5)	34 (51.5)	
Occupational status	With job	61 (47.3)	32 (47.1)	36 (52.9)	0.327
	Without job	68 (52.7)	33 (54.1)	28 (45.9)	
Physical activity	Less than once a week	38 (29.5)	20 (52.6)	18 (47.4)	0.432
	Once a week	47 (36.4)	25 (53.2)	22 (46.8)	
	More than once a week	44 (34.1)	20 (45.5)	24 (54.5)	
Weight		71.33 ± 12.29	73.05 ± 12.23	69.59 ± 12.19	0.870
Height		1.73 ± 0.11	1.74 ± 0.11	1.72 ± 0.12	0.082
BMI		23.73 ± 2.59	24.13 ± 2.60	23.32 ± 2.53	0.120
Perceived stress (PSS)		25.33 ± 6.76	25.82 ± 7.24	24.83 ± 6.25	0.391
Emotional wellbeing (WEMWBS)		43.83 ± 7.82	43.42 ± 8.20	44.25 ± 7.46	0.408
State anxiety (STAI)		35.02 ± 12.52	32.69 ± 12.12	37.39 ± 12.56	0.472
Trait anxiety (STAI)		39.05 ± 11.49	40.74 ± 12.37	37.34 ± 10.33	0.069

Quantitative variables are presented as mean and standard deviation. Qualitative variables are presented as frequency and percentage. PSS, The Perceived Stress Scale; WEMWBS, Warwick-Edinburgh Mental Wellbeing Scale; STAI, State Anxiety Inventory.

### 3.1 Perceived stress

**Figure 2:**
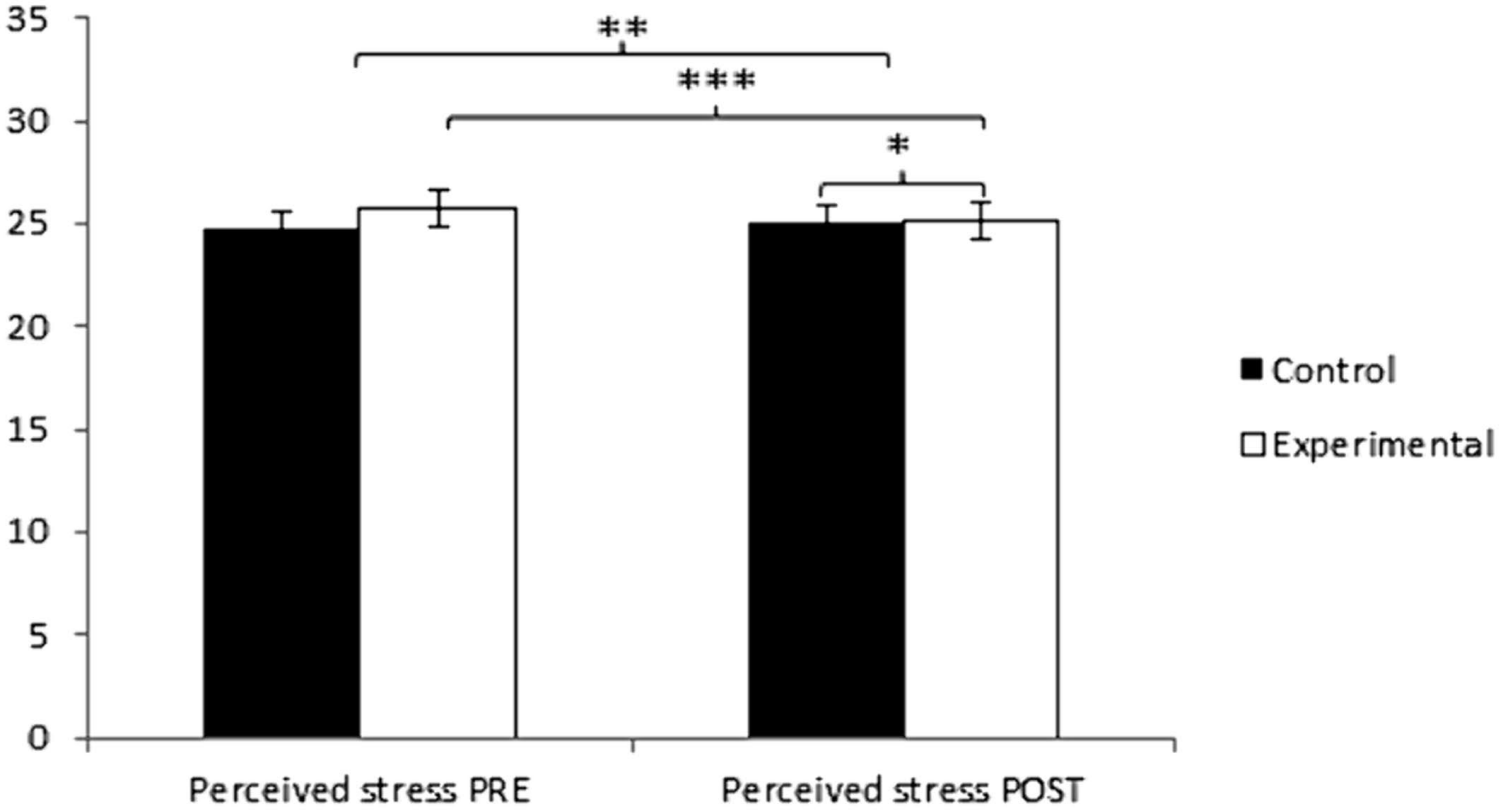
Inter- and intra-group comparisons regarding perceived stress. *p < 0.05. **p < 0.01. ***p < 0.001.

Before the intervention, participants in the Experimental Group (EG) reported higher values in the primary variable of perceived stress (25.82 ± 7.24) compared to those in the Control Group (CG) (24.83 ± 6.25). However, post-measurement revealed higher stress levels in the CG (25.09 ± 6.41) compared to the EG (22.77 ± 6.45). Significant differences were found in Group × Time: *F*(1, 127) = 16.853, *p* = 0.001, η^2^ = 0.117, and in Time: *F*(1, 127) = 11.880, *p* = 0.001, η^2^ = 0.086, but not in Group: *F*(1, 127) = 0.376, *p* = 0.541, η^2^ = 0.003 (Figure 2). The comprehensive analysis showed statistically significant differences between groups in the post-intervention measurement, *t*(127) = 2.053, *p* = 0.042, with a small effect size (*d* = 0.36). Additionally, significant differences were observed in pre and post-measurements for the group undergoing yoga treatment/training, *t*(64) = 3.921, *p* = 0.001, with a small effect size (*d* = 0.44). After Bonferroni correction, the differences remained significant: *t*(127) = 2.053, *p* = 0.042* (adjusted), *d* = 0.36 (small effect size) for group comparison, and *t*(64) = 3.921, *p* = 0.001* (adjusted), *d* = 0.44 (small effect size) for pre-post comparisons. (*Significant after Bonferroni correction, *p* < 0.05/3 = 0.017) (Table 2).

**Table 2:** Effects of yoga program on perceived stress, emotional wellbeing, state anxiety and trait anxiety.

	Pre-intervention			Post-intervention			Group			Time					Group × Time			
	EG	CG	Valor p	EG	CG	Valor p	*F*(1.127)	*P*-value	η^2^	Cohen’s d (Group)	F	*P-*value	η^2^	Cohen’s d (Time)	*F*(1.127)	*P*-value	η^2^	Cohen’s d (Group × Time)
											(1.127)							
Perceived stress (PSS)	25.82 ± 7.24	24.83 ± 6.25	0.58	22.77 ± 6.45	25.09 ± 6.41	0.042	0.376	0.541	0.003	0.36	11.880	0.001	0.086	0.61	16.853	0.001	0.117	0.73
Emotional wellbeing (WEMWBS)	43.42 ± 8.20	44.25 ± 7.46	0.62	47.00 ± 7.10	44.06 ± 7.55	0.14	0.751	0.388	0.006	0.08	9.232	0.003	0.068	0.47	11.383	0.001	0.082	0.598
State anxiety (STAI)	32.69 ± 12.12	37.39 ± 12.56	0.15	28.42 ± 10.26	37.05 ± 12.75	0.001	11,246	0.001	0.000	0.75	11.053	0.001	0.080	0.59	8.008	0.005	0.059	0.50
Trait anxiety (STAI)	40.74 ± 12.37	37.34 ± 10.33	0.30	31.95 ± 9.56	36.19 ± 10.38	0.066	0.066	0.798	0.001	0.42	24.248	0.000	0.182	0.94	16.634	0.001	0.116	0.72

Quantitative variables are presented as mean and standard deviation. Qualitative variables are presented as frequency and percentage. PSS, The Perceived Stress Scale; WEMWBS, Warwick-Edinburgh Mental Wellbeing Scale; STAI, State Anxiety Inventory.

### 3.2 Emotional wellbeing

**Figure 3:**
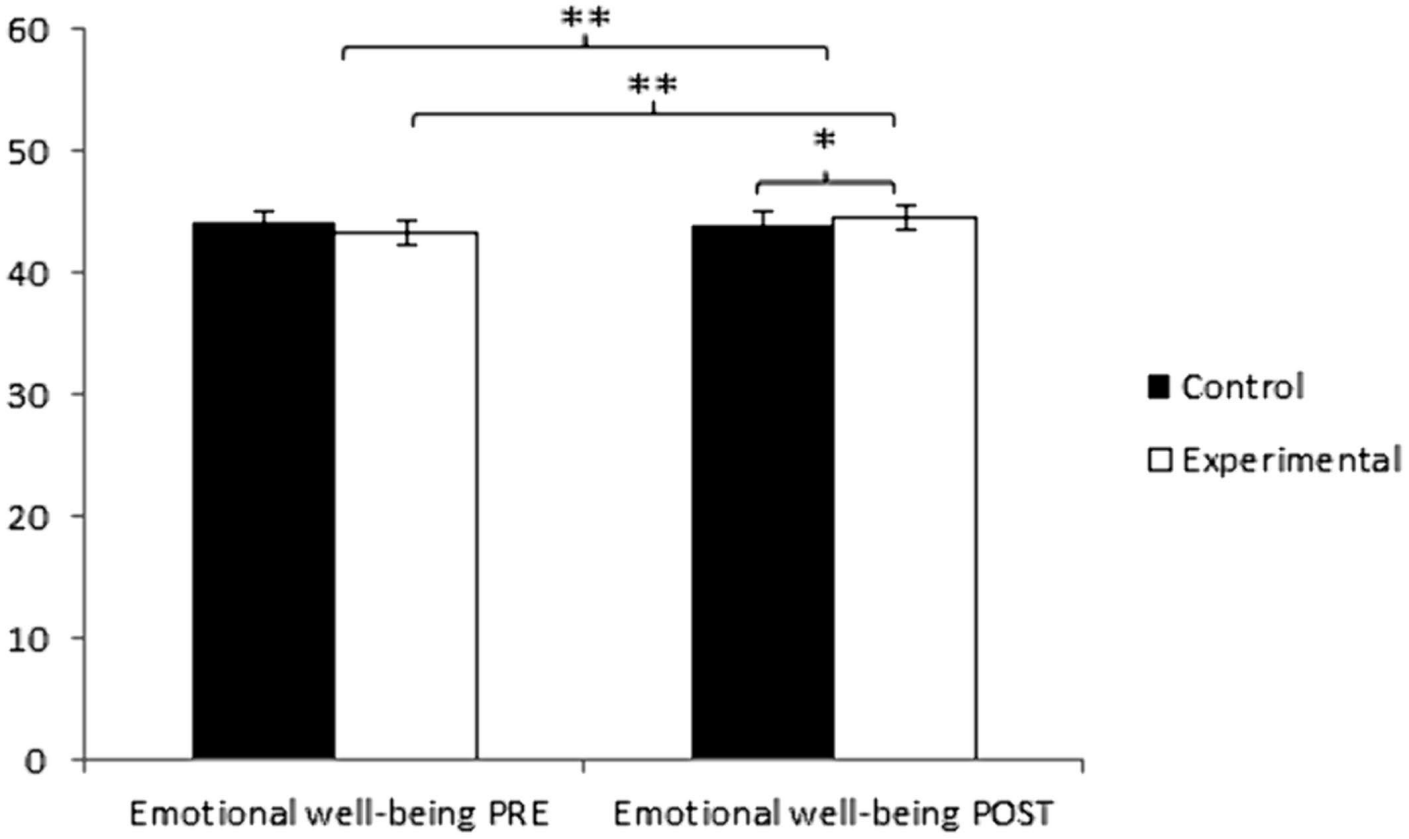
Inter- and intra-group comparisons regarding emotional wellbeing. *p < 0.05. **p < 0.001.

In emotional wellbeing, participants in the Control Group (CG) initially reported higher values (44.25 ± 7.46) compared to those in the Experimental Group (EG) (43.42 ± 8.20). However, conversely, the EG participants (47.00 ± 7.10) achieved higher values than the CG (44.06 ± 7.55). Significant differences were observed in Group × Time: *F*(1, 127) = 11.383, *p* = 0.001, η^2^ = 0.082, and in Time: *F*(1, 127) = 9.232, *p* = 0.003, η^2^ = 0.068, but not in Group: *F*(1, 127) = 0.751, *p* = 0.388, η^2^ = 0.006 (Figure 3). After Bonferroni correction, the comprehensive analysis revealed statistically significant differences between both groups in the post-intervention measurement, *t*(127) = −2.277, *p* = 0.024, with a small effect size (*d* = 0.08). Additionally, statistically significant differences were observed between the pre and post-measurements in the group that received treatment/training in yoga, *t*(64) = −3.405, *p* = 0.001, with a small effect size (*d* = 0.47) (Table 2).

### 3.3 Anxiety

**Figure 4:**
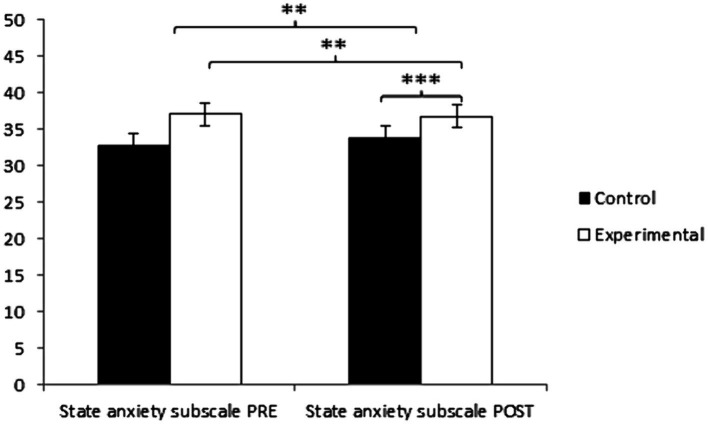
Inter- and intra-group comparisons regarding state anxiety. **p < 0.01. ***p < 0.001.

Regarding state anxiety, participants in the Control Group (CG) reported higher values (37.39 ± 12.56) than those in the Experimental Group (EG) (32.69 ± 12.12) before the start of the intervention, as well as in the post-intervention measurement (37.05 ± 12.75 vs. 28.42 ± 10.26), and significant differences appeared in Group × Time: *F*(1, 127) = 8.008, *p* = 0.005, η^2^ = 0.059, in Time: *F*(1, 127) = 11.053, *p* = 0.001, η^2^ = 0.080, and in Group: *F*(1,127) = 11.246, *p* = 0.001, η^2^ = 0.000 (Figure 4). The comprehensive analysis of the interaction demonstrates the existence of statistically significant differences between both groups in the post-intervention measurement, *t*(127) = 4.240, *p* = 0.001* (adjusted with Bonferroni correction), with a small effect size (*d* = 0.75). Additionally, the existence of statistically significant differences between the pre and post-measurement in the group that received the treatment/training in yoga was observed, *t*(64) = 3.148, *p* = 0.002* (adjusted with Bonferroni correction), with a medium effect size (*d* = 0.38).

**Figure 5:**
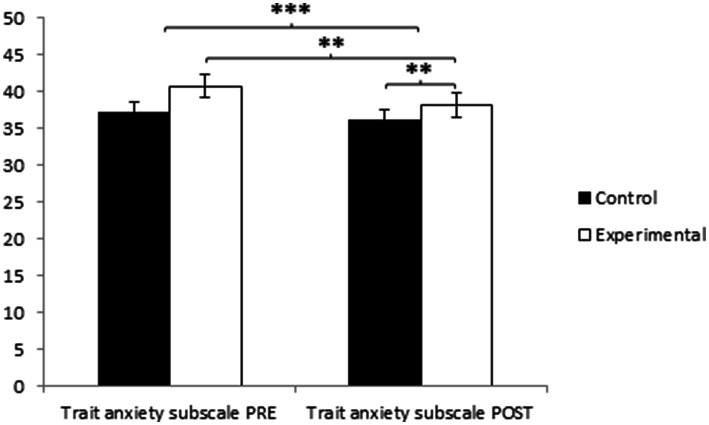
Inter- and intra-group comparisons regarding trait anxiety. *p < 0.05. ***p < 0.001.

Regarding trait anxiety, participants in the Experimental Group (EG) reported higher values (40.74 ± 12.37) than those in the Control Group (CG) (37.34 ± 10.33) before the start of the intervention. Conversely, in the post-measurement, the CG obtained higher values (36.19 ± 10.38) than the EG (31.95 ± 9.56), and significant differences appeared in Group × Time: *F*(1.27) = 16.634, *p* = 0.001, η^2^ = 0.116 and in Time: *F*(1,127) = 24.248, *p* = 0.000, η^2^ = 0.182, but not in Group: *F*(1, 127) = 0.066, *p* = 0.798, η^2^ = 0.001 (Figure 4). The comprehensive analysis of the interaction demonstrates the existence of statistically significant differences between both groups in the post-intervention measurement, *t*(102) = 2.410, *p* = 0.017* (adjusted with Bonferroni correction), with a small effect size (*d* = 0.42). Additionally, the existence of statistically significant differences between the pre and post-measurement in the group that received the treatment/training in yoga was observed, *t*(51) = 6.406, *p* = 0.001* (adjusted with Bonferroni correction), with a medium effect size (*d* = 0.80) (Table 2; Figure 5).

## 4 Discussion

The aim of this study was to analyze the effects of a yoga-based intervention on stress, emotional wellbeing, state anxiety, and trait anxiety in university students. The results of this study provide significant insight into the impact of a 12-week yoga-based intervention on university students. Firstly, regarding perceived stress (primary outcome), a significant decrease was observed in the experimental group (EG) compared to the control group (CG) after the intervention. This aligns with previous studies (33–35), reaffirming the effectiveness of yoga in reducing perceived stress. This is based on physiological processes that include the regulation of the autonomic nervous system (ANS), the peripheral nervous system, the activity of the limbic system, endocrine functions, and inflammatory responses (16); Similarly, it is proposed that yoga has immediate and positive effects on baroreflex sensitivity and heart rate variability (HRV), which results in the reduction of vagus nerve stimulation that in turn leads to a decrease in sympathetic system activation, implying a reduction in the release of stress hormones (36–38).

In terms of emotional wellbeing, a similar trend was observed. Although at the beginning of the study the participants in the control group reported slightly higher levels, those in the experimental group experienced a significant improvement after the intervention. This finding coincides with previous research that has found similar results in populations with comparable characteristics (39, 40). This suggests that the improvement could be related to an increased release of dopamine, which translates into an improved mood, a feeling of happiness and emotional satisfaction, as well as enhanced attention, concentration, and a sense of connection and fullness (41). Furthermore, a decrease in the readiness to take actions is observed, suggesting that practicing mindful meditation could inhibit nervous communication through the glutamate neurotransmitter between the cerebral cortex and the striatum (42).

The results of the present study exhibit notable consistency with previous literature, as evidenced by references (43–45), which underpin the biological basis of anxiety in γ-aminobutyric acid (GABAergic) activity (46). Specifically, a significant reduction in anxiety levels was observed within the experimental group following the implementation of the intervention, in contrast to the stability observed in the control group. These findings underscore the efficacy of the intervention in anxiety reduction, thereby contributing to the understanding and management of this clinical condition.

The implementation of this study within the existing research context reveals fundamental insights into the pathophysiology of anxiety, emphasizing the importance of further exploration in this field. However, it is crucial to acknowledge the methodological limitations inherent in the study design, such as the potential presence of biases or the need for greater sample diversity to generalize the results more broadly. The results of this research underline the importance of considering yoga-based interventions as a viable and effective strategy to promote wellbeing in university students. By offering practical and accessible tools for managing stress and anxiety, these interventions have the potential to significantly improve the university experience and the academic performance of students. Given the nature of the intervention, along with its selection criteria and structural support, no clinically relevant adverse situations were presented. Continuous follow-up of participants allowed for constant communication and timely response to any concerns, as well as the ability to provide additional support when necessary.

The yoga intervention was carried out by a single physiotherapist, which strengthens the internal validity of our results. Variables were measured through validated and self-reported questionnaires, which decreases information bias; likewise, blinding of researchers, assessors, and data analysts prevented the results from being influenced by expectations. Even though the sample size was computed to ensure statistical representativeness, the distinct attributes of the population might constrain the broad applicability of the results. Therefore, it would be beneficial to conduct population-level research linked to educational public policies, as it responds to a problem needing attention worldwide. Future research is needed to determine the optimal dose required to achieve an effective reduction in stress and anxiety, as well as to improve emotional wellbeing. It is also crucial to assess how these results vary at different stages of university life (beginning or end) or during phases of the same academic period (exams, thesis defenses, among others).

Given that preliminary clinical evidence suggests that the effects might not differ from other meditation interventions (47, 48), future research is needed to compare the results between different body–mind activity strategies, as well as to conduct long-term follow-up of these outcomes. This study not only represents a significant step in research on interventions to improve the mental health of university students but also offers valuable information for the development of intervention programs in academic settings. The results obtained can have important implications in promoting wellbeing and reducing stress in the student community, thus contributing to a healthier and more productive university environment. Additionally, it is important to note that in this study, several additional parameters were identified that were initially planned for inclusion in the analysis, but were not presented in this publication. These parameters include D2, MAAS, PAAS and Handgrip Strength. The omission of these parameters represents a deviation from the study protocol. Future research is encouraged to consider including these parameters in subsequent analyzes for a more complete understanding of the observed effects.

## 5 Conclusion

This study highlights the effectiveness of a 12-week yoga-based intervention in reducing perceived stress and anxiety, as well as improving emotional wellbeing among university students, demonstrating the benefits of yoga for stress regulation and mental health. These findings have significant clinical implications, suggesting that yoga can be a valuable complementary therapeutic strategy in managing stress and anxiety in young populations. Furthermore, this research underscores the importance of including yoga programs in academic settings to promote a healthier and more productive university environment. Future research should focus on comparing the efficacy of yoga with other forms of intervention for anxiety and stress and assessing the long-term effects of regular yoga practice. This will not only expand our understanding of yoga-based interventions but also inform the development of policies and programs aimed at improving the mental and physical wellbeing of university students.

## Data availability statement

The raw data supporting the conclusions of this article will be made available by the authors, without undue reservation.

## Ethics statement

This study was approved by the Ethics Committee of the Mid-Atlantic University (CEI/05–006). The studies were conducted in accordance with the local legislation and institutional requirements. The participants provided their written informed consent to participate in this study. Written informed consent was obtained from the individual(s) for the publication of any potentially identifiable images or data included in this article.

## Author contributions

YC-C: Conceptualization, Writing – original draft. MC-F: Supervision, Writing – review & editing. AA-A: Methodology, Writing – original draft. YR-C: Formal analysis, Writing – original draft. AG-M: Conceptualization, Writing – review & editing.
